# Disrupted sleep and associated factors in Australian dementia caregivers: a cross-sectional study

**DOI:** 10.1186/s12877-020-01726-1

**Published:** 2020-08-27

**Authors:** Aisling Smyth, Lisa Whitehead, Eimear Quigley, Caroline Vafeas, Laura Emery

**Affiliations:** 1 School of Nursing & Midwifery, Edith Cowan University, 270 Joondalup Drive, Joondalup, WA 6027 Australia; 2 School of Arts and Humanities (Psychological Services Centre), Edith Cowan University, Joondalup, Australia

**Keywords:** Carers, Caregivers, Sleep, Mood, Psychological well-being, Dementia

## Abstract

**Background:**

Sleep disturbance is an issue reported by caregivers. Waking at night is a feature of dementia and by proxy, sleep disturbance among caregivers is reported to be high. Little is known about the characteristics of dementia caregivers’ sleep and the factors that may influence sleep disruption.

The purpose of this study was to investigate the sleep characteristics and disturbances of Australian caregivers of a person living with dementia. In addition, it evaluated the psychological wellbeing of caregivers by evaluating associations between mood and sleep in this population.

**Methods:**

This study used a cross-sectional, descriptive, correlation design. Participants were recruited with the assistance of Alzheimer’s Australia, Dementia Australia and targeted social media advertising. In total, 104 adult, primary, informal caregivers of people with dementia participated, completing a questionnaire on demographic characteristics, the Depression, Anxiety and Stress Scale (DASS-21) and the Pittsburgh Sleep Quality Index (PSQI).

**Results:**

In this study, 76% of caregivers were female who had been caring for someone living with dementia on average for 4.8 years. 44% of participants had two or more co-morbidities namely cardiovascular disease, osteoarthritis and diabetes. 94% of participants were poor sleepers with 84% with difficulty initiating sleep and 72% reporting having difficulty maintaining sleep. Overall, psychological distress was common with high levels of moderate to severe depression, anxiety and stress. Global PSQI scores were significantly positively associated with depression and anxiety, with the strongest correlation seen with stress scores. Depression scores were also moderately associated with daytime dysfunction. Stress was identified as a significant predictor of overall sleep quality.

**Conclusions:**

Sleep problems are common within the population of dementia caregivers. Due to the nature and duration of caregiving and the progression of dementia of the care recipient, there is the potential for a decline in the caregivers’ mental and physical health. Caregivers of those living with dementia are more likely to have comorbidities, depression, anxiety and stress. Sleep quality is correlated with emotional distress in dementia caregivers although the direction of this association is unclear. Therefore, sleep and psychological wellbeing may be intertwined, with improvements in one aspect resulting in a positive impact in the other.

## Background

Dementia is an inclusive term used to describe a number of neurological conditions resulting in cognitive impairment and can include Alzheimer’s Disease (senile dementia), frontotemporal dementia, vascular dementia, Lewy Body dementia, Korsakoff syndrome (alcohol related brain injury) and younger onset dementia [1–3]. Globally, the number of individuals with a formal dementia diagnosis, rose from 20.2 million in 1990 to 43.8 million in 2016 [4]. A further 9.9 million new cases of dementia worldwide are predicted each year [5] as populations around the globe continue to live longer [6]. These figures could be very much under estimated as it is widely accepted that between 50 and 80% of people affected have no formal dementia diagnosis [7]. This growing epidemic effects not only the individuals living with dementia, but also their families caring for them, the communities they live in and the health care systems they rely upon.

Family or friends are often key informal caregivers for people living with dementia [8, 9]. Globally, over 80 billion hours of informal care is provided to people living with dementia [5, 10]. Whilst providing this care can be highly rewarding, it has also been described as a chronic stressor with caregivers reporting low quality and quantity of sleep [11] and high levels of stress and depression [12]. In fact, sleep disruption is prevalent amongst dementia caregivers with over 90% of caregivers experiencing sleep disturbances [13].

The National Sleep Foundation recommend that older adults (≥65 years) require seven to 8 h sleep for optimal physical and psychological wellbeing [14]. Yet, numerous studies have illustrated that dementia caregivers sleep significantly less than that with estimates reporting most caregivers sleep less than 7 h per night [15]. Not only are caregivers sleeping less than they should, the sleep they do get is of significantly lower quality than their non-caregiver counterparts [11].

Poor sleep is associated with a myriad of negative physical and psychological outcomes including hypertension, obesity, mood disorders and dementia [16]. A disrupted sleep pattern is also recognised as a significant factor in predicting caregiver strain [17, 18] and perhaps more importantly, in predicting placing an individual into long term care [19]. Enabling people living with dementia to stay at home, rather than transfer to long-term care is the optimal outcome for many families. However, this cannot be to the detriment of the caregiver’s own physical and/or psychological wellbeing. Therefore, in order to support the person living with dementia (PLWD) to remain in the community, maintaining caregiver health is vital. Given the pivotal role sleep has in a myriad of physiological processes, it is essential to optimise and preserve caregivers sleep.

Despite the important role sleep plays in dementia caregiver health, it remains an understudied population, particularly within the Australian context. In fact, a recent report by Carers Australia (2019) identified no Australian studies on sleep disruption in dementia caregivers. Only sixteen international studies were identified which subjectively measured sleep in the dementia caregivers population, with the majority of studies not reporting on the causes or consequences of disturbed sleep [15].

In order to address this paucity of Australian data, the purpose of this study was to elucidate the sleep characteristics and disturbances of Australian caregivers of PLWD. Furthermore, this study will determine whether there is a relationship between sleep and psychological wellbeing among caregivers of community-dwelling people living with dementia. Lastly, we will aim to identify significant predictors of poor sleep, which in turn could offer a therapeutic target of poor sleep in dementia caregivers.

## Methods

### Recruitment

This study used a cross-sectional, descriptive, correlation design. One hundred and four (104) informal caregivers of people with dementia living in the community were enrolled in the study. Participants were invited via a number of organisations including Alzheimer’s Australia, Dementia Australia and targeted social media advertising. Mail outs were conducted and online questionnaires distributed. The inclusion criterion required the participant be an adult (18 years or older), primary, informal caregiver of a community-dwelling person living with dementia. Power analysis was undertaken to compute minimum number of sample size required. A sample size of at least 50 participants is necessary to detect a medium to large effect, with 80% power, assuming a two-tailed t-test.

### Ethics

This study was granted ethical approval by Edith Cowan University Human Research Ethics Committee (No. 18438). Informed written consent was obtained from all participants prior to participation. Participants received no incentive for taking part.

### Materials

The survey collected demographic characteristics including gender, age, body mass index (BMI), as well as information about pre-existing medical history, caregiving history and use of respite. The survey also included the 21 questions from the Depression, Anxiety and Stress Scale (DASS-21) [20] and the Pittsburgh Sleep Quality Index (PSQI) [21].

The DASS-21 is a self-report questionnaire assessing levels of caregiver stress, anxiety and depression over the previous seven [7] day period. The results provide an indication of the individual’s perception of their experience of depression, anxiety and stress. The DASS-21 is often used as a screen for high levels of distress when the depression and/or anxiety scores are high, and as a screen for the presence of a significant life event or problem if the stress score is elevated. DASS scores correspond to ranges of severity (normal, mild, moderate, severe, extremely severe) for depression, anxiety and stress individually [20].

The PSQI [22] is a self-report questionnaire assessing levels of perceived quality and patterns of sleep over the previous month across seven components (subjective sleep quality, sleep latency, sleep duration, habitual sleep efficiency, sleep disturbances, use of sleep medication and daytime dysfunction). Each component has a potential score of three, with a higher score indicating poorer sleep related performance. The total score for the seven components creates a global score (range 0 to 21) where a score greater or equal to five (≥5) indicates that the person is a “poor” sleeper, with severe difficulties in at least two of the seven components or moderate difficulties in three or more components [22].

Presence of comorbidities was determined by participants having one or more chronic conditions. As per World Health Organisation (WHO), chronic conditions were identified as those requiring ‘ongoing management over a period of years or decades’ covering a wide range of health problems such as heart disease, diabetes, asthma, immunodeficiency disorder, depression and schizophrenia.

### Statistical analyses

Data analysis was undertaken using IBM SPSS (Version 25) and GraphPad Prism 7. Descriptive statistics were generated for demographic characteristics, DASS-21 and PSQI scales Spearmans rho (*r*) correlation analysis was used to assess the relationship between categorical components of DASS-21 (depression, anxiety and stress) and both PSQI global scores and individual component scores (subjective sleep quality, sleep latency, sleep duration, sleep efficiency, sleep disturbances, use of sleeping medication and daytime dysfunction). For logistic regression, all variables were categorical (age in years, gender, BMI, length of care in months, comorbidities, DASS-21 component scores. An *r* of 0.3 was considered a medium correlation and an r of 0.5 was considered a large correlation [23]. Non-paired *t*-tests were used to determine if these values were statistically significant. Results were considered statistically significant if *p* < 0.05. Multivariate stepwise regression analysis was undertaken to determine the predictive factors of global PSQI scores and identify variables significantly associated with sleep quality. Independent variables included in multivariate stepwise regression analyses were age, gender, BMI, length of care, comorbidities and DASS-21 subscale scores Subjects with missing data on either PSQI or DASS-21 were excluded from inclusion in correlation and regression analyses.

## Results

### Characteristics of the caregivers

One hundred and four (*n* = 104) surveys were completed in either hardcopy (*n* = 49) or online (*n* = 55) (Table 1). As the surveys were widely distributed by service providers and links made available online, the response rate cannot be determined.

**Table 1:** Demographic of participants

Characteristic	*n*	% or mean+/− SD
Gender		
Male	25	24%
Female	79	76%
All	104	100%
Age	104	67.6 ± 10.2
BMI (kg/m^2^)	99^a^	27.9 ± 5.8
Caregiver role (Months)	94^b^	58.4 ± 61.1
Comorbidities		
None	39	37.5%
One	19	18.3%
Two +	46	44.2%
Total	104	100%
Use of Respite		
Yes	8	8.3%
No	88	91.7%
Total	96^c^	100%

^a^ *n* = 5 missing;^b^ *n* = 10 missing;^c^ *n* = 8 missing

Participating caregivers were predominantly female (*n* = 79; 76%) with a mean age of 67 years (Range 61–74 years). The average BMI for respondents was 27.9 kg/m^2^ (range 15.7–51.0 kg/m^2^) (Table 1) which lies within the overweight category. While half of the participants (*n* = 52; 50%) had a BMI within the healthy weight range (18.5–24.9), twenty two participants (21%) were classified as underweight (BMI less than 18.5) and 30 participants (29%) were classified as overweight. The average length of time in the caregiving role in this study was 58 months (4.8 years), ranging from 5 months to 35 years. Only 8% (*n* = 8) of participants reported using formal respite services and all were female.

38% (*n* = 39) of participants reported no comorbidities, 18% of participants (*n* = 19) reported one comorbidity and 44% (*n* = 46) reported two or more comorbidities. The most commonly reported comorbidities were cardiovascular disorders (*n* = 32) (mainly hypertension and hypercholesterolemia), bone and joint disorders (*n* = 14) (mainly osteoporosis and arthritis) and endocrine disorders (*n* = 16) (mainly pre-diabetes, diabetes and thyroid dysfunction).

### Subjective Sleep quality

The majority of participants (*n* = 97, 94%) reported a global sleep score equal or greater than 5 which indicated they had clinically significant sleep issues in the preceding month with a mean global score of 9.1 ± 3.7 (Table 2). The highest scoring individual subcomponents included sleep latency (1.6 ± 1.0), subjective sleep quality (1.5 ± 0.7) and sleep disturbances (1.5 ± 0.6). 84% (*n* = 60) of participants had a sleep latency period greater than 15 min, indicative of issues initiating sleep (Table 2). 72% of participants had sleep efficiencies less than 85% suggesting issues with maintaining sleep while in bed. 34% of participants had taken sleep medication at least once a week over the previous month. Participants were invited to add any additional relevant comments to their PSQI sleep assessment. Forty participants provided additional information regarding their sleeping issues and identified issues which fell broadly into three themes: Sleep disruption due to caregivers physical needs such as pain and restless legs (35%), caregivers emotional distress such as stress, anxiety and worrying (30%) and responding to care recipient needs (35%).

**Table 2:** Characteristics of Caregivers’ sleep

Measure	***n*** (%)	PSQI Component Mean +/− SD
**Global Score**	71 (100%)	9.12 ± 3.7
**Subjective Sleep Quality**	71 (100%)	1.5 ± 0.7
Very Good	3 (4.2)	
Fairly Good	34 (47.9)	
Fairly Bad	28 (39.4)	
Very Bad	6 (8.5)	
**Sleep Latency**	71 (100%)	1.6 ± 1.0
≤ 15 mins	11 (15)	
16–30 min	20 (28.2)	
31–60 min	26 (36.6)	
60+ mins	14 (19.7)	
**Sleep duration**	71 (100%)	1.2 ± 0.9
> 7 h	14 (20)	
6–7 h	36 (51)	
5–6 h	11 (15)	
< 5 h	10 (14)	
**Sleep Efficiency**	70 (100%)	1.4 ± 1.1
≥ 85%	19 (27.5)	
84–75%	19 (27.5)	
74–65%	15 (21.5)	
< 65%	17 (24.5)	
**Sleep Disturbances**	71 (100%)	1.5 ± 0.6
1	39 (54.9)	
2	28 (39.4)	
3	4 (5.6)	
**Daytime Dysfunction**	71 (100%)	1.1 ± 0.8
0	15 (21.1)	
1	40 (56.3)	
2	12 (16.9)	
3	4 (5.6)	
**Frequency of Sleeping Medication**	71 (100%)	0.8 ± 1.1
Never	47 (45)	
Once per week	5 (7)	
Twice per week	9 (13)	
Three + per week	10 (14)	

### Depression, anxiety and stress in caregivers

21% of participants reported mild depression scores, 11% of participants reported mild anxiety scores and 21% of participants reported mild stress scores. Over a third of respondents reported moderate to extremely severe stress levels (*n* = 26, 37%) and depression (*n* = 25, 35%) and more than a quarter (*n* = 20, 28%) of respondents reported moderate to extremely severe anxiety levels (Table 3).

**Table 3:** Depression, anxiety and stress in caregivers (DASS-21)

Clinical classification	Depression ***n*** (%)	Anxiety ***n*** (%)	Stress ***n*** (%)
Total	71 (100%)	71 (100%)	71 (100%)
Normal	31 (43.7)	43 (60.6)	29 (41.4)
Mild	15 (21.1)	8 (11.3)	15 (21.4)
Moderate	14 (19.7)	10 (14.1)	18 (25.7)
Severe	6 (8.5)	4 (5.6)	6 (8.6)
Extremely Severe	5 (7.0)	6 (8.5)	2 (2.9)

In the bivariate analyses, numerous subcomponents of the PSQI scale were significantly associated with measures of depression, anxiety and stress (Table 4). The global PSQI score is significantly positively associated with depression (*r* = .24), anxiety (*r* = .28) and stress scores (*r* = .41). Stress scores also significantly correlated with other PSQI subcomponents including subjective sleep quality (*r* = .36), sleep latency (*r* = .30) and daytime dysfunction (*r* = .40). Depression scores were moderately associated with daytime dysfunction (*r* = .48).

**Table 4:** Correlation between PSQI sub-component scores and DASS-21 sub-component score in dementia caregivers

PSQI component	DASS-21 Depression	DASS-21 Anxiety	DASS-21 Stress
PSQI Global Score	0.24*	0.28*	0.41**
Subjective sleep quality	0.30*	0.20	0.36*
Sleep latency	0.26*	0.18	0.30*
Sleep duration	−0.11	−0.06	0.02
Sleep efficiency	0.07	−0.01	0.22
Sleep disturbances	0.21	0.30*	0.29*
Use of sleeping medication	0.03	0.29*	0.24*
Daytime dysfunction	0.48**	0.33*	0.40**

*p < 0.05, **p < 0.001

### Predictors of Sleep quality in caregivers

To understand the relationship between the predictors and global PSQI scores, multivariate stepwise regression analyses were conducted to assess variables significantly associated with sleep quality. The dependent variable was the global PSQI score and the independent variables were age, gender, BMI, use of respite, length of care, comorbidities and scores on the sub-scales of DASS-21 scale (Depression, Anxiety and Stress). Stress was the only significant covariate of global PSQI scores. Stress scores could statistically significantly predict PSQI Global scores, accounting for 18% of variance. The standardised coefficient was .425, which was statistically significant (*p* < 0.001), as was the overall model (F = 14.2, *p* < 0.001). All other variables were excluded from the model due to non-significance. Forward stepwise regression analyses were then undertaken with global PSQI scores as the dependent variable and stress scores as a predictor while adjusting for the following confounders: gender, age, comorbidities of the caregiver and length of care. While these confounders were not statistically significant, they were retained within the model to adjust the effects of stress on PSQI. After adjusting for these factors, that model remains statistically significant (F = 3.86, *p* = .004), and accounts for 17% of the (adjusted) variance in sleep scores. For every one unit increase in stress scores, PSQI Global scores increases by a score of 1.27 (t = 3.553, *p* = .001). This regression model confirms that stress is a key, significant covariate of self-reported sleep issues.

## Discussion

A recent Australian report highlighted a significant gap in the literature around Australian caregivers of a PLWD [15]. Our study provides a comprehensive overview of Australian dementia caregivers sleep characteristics, associations between psychological wellbeing and sleep and highlights a predictive role for stress in sleep quality. All previous international studies identified an average PSQI >/ 5 in dementia caregiver studies [15] highlighting the prevalence of the issue. As expected, and consistent with previous studies [13], Australian caregivers of PLWD have a high prevalence of poor sleep with 94% of participants classified as poor sleepers. The PSQI global score (9.12 ± 3.7) was higher than those found in some dementia caregiver studies [24, 25] but comparable with others [13]. The PSQI sub-components which greatest contributed to the overall score were sleep latency (time taken to fall asleep), sleep quality (overall subjective quality) and sleep disturbances. Sleep latency and sleep disturbances have previously been identified as the most common contributors to sleep quality in caregivers of PLWD in a recent systematic review [11]. Given the dearth of Australian specific data, this study identified important depth of detail around caregiver’s sleep characteristics. 85% of caregivers took longer than the recommended 15 min to fall asleep, only 20% of caregivers slept more than 7 h, 72% of caregivers had sleep efficiency lower that the recommended 85 and 55% of caregivers used sleep aiding medication in the previous month. Taken together, these descriptive findings present a novel and ominous overview of the poor sleep health conditions that Australian dementia caregivers experience.

Caregivers of community dwelling people living with dementia reported poor sleep quality and high levels of depression, anxiety and stress. This study found that poor sleep was correlated with subjective feelings of depression, anxiety and stress, which were in keeping with previous literature around caregiving and psychological distress. More than half of the caregivers surveyed reported symptoms of depression and stress (56 and 58% respectively) and 39% reported anxiety. These findings are in keeping with that of a recent study of dementia caregivers in rural Victoria, where 49% of caregivers reported depression or stress and 26% reported anxiety [12].

Previous work has identified an association between depression and poor sleep among dementia caregivers [26, 27]. Reduced quality of sleep and depression are higher among caregivers of people living with dementia [17]. Furthermore, caregivers of people living with dementia, who were also depressed, experience a greater variation in sleep patterns [17].

This study reveals sleep scores were significantly associated with measures of depression, anxiety and stress. These findings illustrate the interplay between sleep quality and quantity and psychological wellbeing in caregivers providing care for an individual living with dementia. Further exploration of predictive factors in caregivers sleep quality identified stress as a key predictor of poor overall sleep quality whilst adjusting for age, gender, comorbidities and length of care. This is a novel finding and represents a potential therapeutic target to improve sleep quality in dementia caregivers.

Despite the high prevalence of self-identified poor sleep, overnight respite was only used by 8% of participants, which is in keeping with previous research that cites 87% of Australian caregivers have never used respite services [28]. Access to respite care remains one of the major means of easing caregiver burden and is frequently identified as needed by caregivers, yet remains underutilised. Whilst a high proportion of dementia caregivers report a need for respite services, there is insufficient awareness of and access to respite services for caregivers [29]. Respite service availability can be invaluable with caregivers reporting lower stress levels and improved health after use [30]. Sleep disturbances have also been shown to be partially reversed for caregivers during periods of respite care [31], perhaps due to reversal of hyper stressed state. Respite services have been cited as an important measure to allow caregivers time to attend to their own health, imperative to supporting the caregiver to continue in providing care for their loved one [30].

A recent Australian parliamentary inquiry into sleep health identified sleep as a foundation of positive health and wellbeing, alongside diet and exercise [32]. Furthermore, the report urged government to prioritise sleep health as a national priority given its pivotal role in maintaining health. It is clear that caregivers of PLWD have sub-optimal sleep which is associated with poorer psychological wellbeing and potential increased risk of developing chronic health conditions such as diabetes and cardiovascular disease [16]. Interestingly, cardiovascular disease and endocrine disease such as diabetes were the most prevalent comorbidities in our study population. Previous studies have also identified high blood pressure, diabetes and arthritis as the most prevalent chronic disease in the dementia caregiver group which is in keeping with our findings [33].

Managing sleep and its associated mediators will have direct impact on both the caregiver’s own health as well as the caregiver/ care recipient relationship. It is imperative to provide educational support around sleep, consider practical interventions such as overnight respite and to address stress management interventions for caregivers, in turn, reinstating and preserving sleep of this critical population of informal caregivers.

### Limitations

A limitation of this study was the small sample size despite numerous attempts to recruit participants and involvement of national organisations. Although over 100 participants provided demographic details, only 71 participants completed all questionnaires and were included in statistical analyses. Challenges related to recruiting caregivers and particularly caregivers of people living with dementia has been described in numerous publications [12, 30, 34, 35]. Caregivers of people with dementia living in the community are more likely to be female and this was represented in our population. Furthermore, caregivers who are under the greatest stress may also be those least likely to participate. However, this remains one of the larger studies of Australian dementia caregivers sleep. Another limitation within this study was that no data was collected regarding the severity of dementia or behavioural disturbances, which may impact sleep of the caregiver.

## Conclusion

Sleep problems are widespread within the population of dementia caregivers. Given that the majority of people living with dementia are reliant on family caregivers, minimising health and psychological impact on caregivers should be of major concern. Furthermore, sleep quality is correlated with emotional distress in dementia caregivers, so improving the sleep of caregivers may in turn improve their psychological wellbeing. Conversely, stress is a significant predictor of poor sleep, so managing stress may have a positive impact on sleep.

Dementia caregivers are often older, with co-existing morbidities and high levels of psychological distress. With prolonged caregiving and the progression of dementia of the care recipient, there is the potential for a concurrent decline in the caregivers mental and physical health. In order to support the caregiver in their role, it is of the upmost importance that we promote and maximises their health and wellbeing. By managing and minimising the negative factors associated with caregiving, we can enhance caregiving satisfaction and gratification. In turn, this can ensure optimal caregiver-care recipient relationship, supporting the person living with dementia to remain at home as long as possible.

## Data Availability

The datasets used and/or analysed during the current study are available from the corresponding author on reasonable request.

## Abbreviations

DASS-21: Depression, Anxiety and Stress Scale-21
PLWD: Person/People living with dementia
PSQI: Pittsburgh Sleep Quality Index
WHO: World Health Organisation
